# Regulation of Retinoid Receptors by Retinoic Acid and Axonal Contact in Schwann Cells

**DOI:** 10.1371/journal.pone.0017023

**Published:** 2011-02-28

**Authors:** Maria-Jesus Latasa, Jose Miguel Cosgaya

**Affiliations:** Department of Endocrine and Nervous System Physiopathology, Instituto de Investigaciones Biomédicas, Consejo Superior de Investigaciones Científicas, Universidad Autónoma de Madrid, Madrid, Spain; National University of Singapore, Singapore

## Abstract

**Background:**

Schwann cells (SCs) are the cell type responsible for the formation of the myelin sheath in the peripheral nervous system (PNS). As retinoic acid (RA) and other retinoids have a profound effect as regulators of the myelination program, we sought to investigate how their nuclear receptors levels were regulated in this cell type.

**Methodology/Principal Findings:**

In the present study, by using Schwann cells primary cultures from neonatal Wistar rat pups, as well as myelinating cocultures of Schwann cells with embryonic rat dorsal root ganglion sensory neurons, we have found that sustained expression of RXR-γ depends on the continuous presence of a labile activator, while axonal contact mimickers produced an increase in RXR-γ mRNA and protein levels, increment that could be prevented by RA. The upregulation by axonal contact mimickers and the transcriptional downregulation by RA were dependent on *de novo* protein synthesis and did not involve changes in mRNA stability. On the other hand, RAR-β mRNA levels were only slightly modulated by axonal contact mimickers, while RA produced a strong transcriptional upregulation that was independent of *de novo* protein synthesis without changes in mRNA stability.

**Conclusions/Significance:**

All together, our results show that retinoid receptors are regulated in a complex manner in Schwann cells, suggesting that they could have a prominent role as regulators of Schwann cell physiology.

## Introduction

*All-trans*-retinoic acid (RA) and other retinoids are potent regulators of morphogenesis, cell growth and differentiation, especially in the nervous system, where their abundance is comparatively very high [1]. RA induces neurite outgrowth and neuronal differentiation of several neural cell types, including embryonic and adult stem cells, dorsal root ganglia neurons (DRGN), or neuroblastoma cells [2], [3], [4]. In PC12 cells, RA inhibits cell proliferation and mimics the effect of neurotrophins on the expression of several genes, although the retinoid does not cause neurite extension in parental PC12 cells [5], [6], [7]. In contrast, RA can stimulate neuritogenesis in cAMP-dependent protein kinase A deficient PC12 cell lines that express high levels of RA receptor [8].

Most of the studies on RA functions on the nervous system relate to its function as a morphogen during neural tube development or as a differentiation factor for central nervous system (CNS) neurons, but RA signaling also occurs in the peripheral nervous system (PNS). In the sciatic nerve of adult rats, all the required components of the RA signaling pathway (retinoid X receptors, RA receptors, RA synthesizing enzymes RALDH-1, -2 and -3, as well as the cellular retinoid binding proteins CRBP-I, CRABP-I and -II) are expressed [9]. During regeneration after nerve crush or transection, local production and activation of RA in SC is greatly increased [9], [10], suggesting a potential role for RA during PNS development and repair. Moreover, the fact that retinoid receptors seem to be present on SC [11] suggests that RA could also play a role in SC physiology during development or in pathological conditions during Wallerian degeneration and remyelination, and not only on axonal growth and regeneration. Furthermore, RA has been shown to exert a variety of functions on SC, including the induction of neuregulin receptor erbB3 [10], cooperation with BMP2 to induce GDNF [12], or downregulation of CNTF expression [13].

On the other hand, RA has been shown to induce differentiation of neuroblastoma cells towards a Schwann cell-resembling phenotype [14]. It is also a major part of a differentiation cocktail capable of inducing Schwann cell differentiation from neural stem cells [15], bone-marrow stromal cells [16], adipose tissue-derived stem cells [17] or human umbilical cord-derived mesenchymal stromal cells [18]. Additionally, it has been described that the loss of RA in zebrafish results in a lack of myelination, although RA signaling seems to be required at an early stage of nervous system development, long before the onset of myelin gene expression and even before the formation of overt glial-specific precursors [19]. More recently, we have been able to show that retinoids participate in the regulation of peripheral myelin formation [20]. Both *in vitro* by using a myelinating DRGN/SC coculture system, as well as *in vivo* during the development of the sciatic nerve, RA is a strong inhibitor of PNS myelin formation through two different mechanisms, one involving Egr2 (also known as Krox20) upregulation, which is dependent on retinoid X receptor (RXR) activation, and another one that implies MAG downregulation through binding of the retinoid to retinoic acid receptor (RAR).

The members of the nuclear receptor superfamily act as ligand-dependent transcription factors that regulate the transcription of the genes that contain specific hormone response elements (HREs) in their promoters. The retinoids exert their actions through their binding to two groups of nuclear receptors. *All-trans*-RA binds with high affinity to RARs and, with a lower affinity, to RXRs, each one being codified by three separate genes (-α, -β and -γ). Another retinoid, *9-cis*-RA presents a similar affinity in its binding to both receptors, while the so called rexinoids are able to differentially activate RXRs. RARs function only as heterodimers with RXRs, regulating the transcription of the genes that present retinoic acid response elements (RAREs) in their promoters. Gene regulation by rexinoids through RXR can be mediated by retinoid X response elements (RXREs) present in their promoters to which they bind as RXR-RXR homodimers, as well as by acting as heterodimers of RXR in combination with several members of the nuclear receptor superfamily [21].

In addition to the concentration of retinoids and rexinoids, the ability of a particular cell type to respond to these hormones also depends on the relative expression levels of the different receptors present at this particular cell type. The pleiotropic effects of retinoids in development can partially be explained by the patterns of expression of the various RAR and RXR genes [22]. Nuclear receptor expression is under the control of several factors, and, among others, the retinoids are one of the most important. For instance, RA is a potent regulator of RAR-β expression in different cell types, both of neural and non-neural origin [6], [23], [24], [25], although there are also descriptions of regulation of RAR-α by estrogen [26], RAR-β and RXR-β by interleukin-1 [27], RAR-β by nerve growth factor [28], as well as RAR-α and -γ by β-carotene [29].

As SC differentiation is under the control of retinoids, we sought to investigate if retinoid receptors levels were also regulated in this cell type during its differentiation, as well as by the retinoid itself. Using both purified SC cultures, as well as myelinating cocultures of SCs with sensory DRGNs, we have been able to show that RXR-γ is transcriptionally upregulated by axonal contact, while RAR-β, as well as RXR-γ levels are differentially regulated by RA in SCs. RA produces a strong upregulation of RAR-β, while inhibiting RXR-γ expression.

## Materials and Methods

### Ethics Statement

All animal work was done in compliance with the European Community Law (86/609/EEC) and the Spanish law (R.D. 1201/ 2005). All experimental protocols were approved by the Ethics Committee of the Consejo Superior de Investigaciones Cientificas (SAF2003-02528; SAF2004-05343; SAF2007-61693).

### Rat SC cultures

SCs were isolated and purified as previously indicated [30]. After cytosine arabinoside and Thy-1.1 purification, SCs cultures were plated in poly-L-Lysine-coated plates and expanded in 10% heat-inactivated fetal bovine serum (FBS)-containing Dulbecco's modified Eagle's medium (DMEM), in the continuous presence of 2 µM forskolin (Calbiochem) and 60 µg/ml bovine pituitary extract (BPE; Gibco). Cultures were always used within less than four passages and all experiments were repeated with SCs from different preparations. For experiments, freshly seeded SCs were allowed to attach O.N. in regular media, changed to DMEM containing 10% of newborn bovine serum (NBS) depleted of retinoids (achieved by sequential incubations with AG1-X8 resin and charcoal), in the presence of 2 µM forskolin and 60 µg/ml BPE. When the effect of BPE and/or forskolin was to be analyzed, these factors were removed form the cell culture media already at the time of seeding and the cultures were allowed to adapt to the new conditions for at least 24 hours before adding the different treatments. In any case, controls were always treated with an equivalent amount of vehicle (0.01% ethanol for a typical 1 µM RA treatment) for the same time. The data depicted in Figure 5 is a reverse time-course in with all the samples were processed at the same time, having been treated with the retinoid for the time indicated in the X-axis. Control cultures were treated with vehicle (0.01% ethanol) for the longest time point analyzed (48 hours).

### Rat DRGN/SC Cocultures

Purified DRGN and SC cultures were prepared by using methods previously described [30]. In short, neuronal cultures were established from DRGs obtained from Wistar rat embryos at 15 days of gestation from our animal facility. DRGNs were dissociated and plated onto collagen coated coverslips or plastic 6-well plates. Nonneuronal cells were eliminated by cycling (three 2-day cycles) with a fluorodeoxyuridine-containing medium (10 µM). Nerve growth factor (NGF)-dependent neurons were then maintained for 1 week in medium consisting of 10% FBS in high-glucose minimum essential medium (MEM) and 100 ng/ml of NGF.

SCs were isolated from the sciatic nerves of 4-day-old rat pups as previously described [30]. SCs were purified by using cytosine arabinoside and Thy-1.1 antibody (Sigma) mediated lysis of the fibroblasts. Approximately 100,000 purified SCs were then seeded onto 3-weeks-old purified neuronal cultures of ≈50,000 cells. On contact with the axons, SCs proliferated rapidly until the axons were fully populated. When proliferation ceased, SCs began to elongate and ensheath the axons (premyelination stage). During this stage, media was progressively changed to media containing 10% NBS depleted of retinoids. At this time (≈7 days after seeding), once the SCs have ceased to proliferate because they have completely populated the cultures and already have established a one-to-one relationship with the axons, the cocultures were induced to myelinate (myelination stage) by the addition of ascorbic acid (50 µg/ml), which is necessary for the formation of the basal lamina, an absolute requirement for myelin formation. This allows us to discriminate between the proliferation/premyelination stages and the properly called myelination stage.

Treatments with the different retinoids (all at 1 µM unless otherwise indicated) were initiated at the time of ascorbic acid addition (considered always as day 0 in all the experiments), and replenished with every feeding, together with new ascorbic acid, every 2 to 3 days. Sister control cultures received an equivalent amount of vehicle (0.01% ethanol for the 1 µM RA treatments).

### Western Blot Analysis

Samples were prepared for Western blot analysis by homogenization in radioimmunoprecipitation assay (RIPA) buffer [PBS with 1% Nonidet P-40/0.5% deoxycholate/0.1% sodium dodecyl sulfate (SDS)/1 mM PMSF/Complete protease inhibitor tablets (Roche)] followed by high-speed centrifugation. Protein determination was made by using the BCA™ Protein Assay Kit (Pierce). Equivalent amounts of total protein extract from each sample were electrophoretically separated on 10–15% discontinuous acrylamide gels, transferred to pure nitrocellulose membranes (PROTRAN BA85, Schleicher and Schuell, 0.45 µm), and the equal loading and transfer of the samples was monitored by Ponceau staining of the membranes. The different proteins were visualized by incubation with specific antibodies overnight at 4°C, followed by incubation with a secondary antibody for two hours at room temperature. The rabbit polyclonal antibody against RXR-γ (Anaspec) was used at a dilution of 1∶1,000. Secondary horseradish peroxidase (HRP)-conjugated antibodies (Jackson ImmunoResearch) were used at a dilution of 1∶10,000. The blots were developed by chemiluminescence (Immun-Star™ HRP Chemiluminiscent Kit, Bio-Rad) as indicated by the manufacturer. Blots were quantitated with the imaging and analysis software ImageJ 1.42d (http://rsbweb.nih.gov/ij/).

### Real time quantitative PCR

RNA was extracted using TRI® Reagent (Sigma) and quantitated with a Nanodrop™ spectrophotometer (Thermo Scientific). 1 µg total RNA from each sample was reverse transcribed with iScript cDNA Synthesis Kit (Bio-Rad) and the relative levels of the genes of interest determined by quantitative real time PCR using FastStart Universal SYBR Green Master (ROX) mix (Roche) in a Mx3005P instrument (Stratagene). Every measure was always performed in duplicate (two PCR reactions from the same RT sample) and the average of those technical replicates was considered as the C_t_ for that particular sample. All gene expression levels were normalized to the housekeeping genes 18S rRNA or cyclophilin A.

The primers were designed by using the MacVector suite (http://www.macvector.com/) coupled with Amplify software (http://engels.genetics.wisc.edu/amplify/). Primers were designed from common sequences in the rat and mouse genes and, when possible (multiexonic genes), spanning two consecutives exons to avoid interference from possible genomic DNA contamination. For monoexonic genes, a DNase treatment of the samples was performed prior to cDNA sysnthesis. In all cases, primers were first tested by conventional RT-PCR to produce a single amplicon of the correct size, followed by Q-RT-PCR to avoid primer-dimer artifacts and ensure a lineal range of quantitation. When several mRNA splice variants were present for a given gene, the primers were always designed to recognize all known isoforms. The primers used are indicated in Supp. Info. Table S1.

### Determination of hnRNA

hnRNA levels were also determined by RT-Q-PCR, with the following differences from regular mRNA determination. The samples were always treated with DNase to prevent genomic DNA contamination and reverse transcribed with iScript Select cDNA Synthesis Kit (Bio-Rad) using specific oligonucleotides for the genes of interest as primers (the same reverse primer used in the subsequent PCR). hnRNA levels were determined by using primers from an exon and a contiguous intron (exon 3 and intron III for RAR-β and exon 2 and intron II for RXR-γ), so DNA amplification only could occur from newly formed, immature non-spliced RNA.

### Statistical analysis

Throughout the whole article, data shown is representative of at least two separate experiments performed each time at least in duplicate (experimental replicates). Significance was determined by Student's *t*-test (unpaired) analysis using StatPlus:mac LE2009 coupled with Microsoft Excel:mac 2008. Unless indicated otherwise, all comparisons were made against their respective controls. Throughout the test, differences were considered as significant when p<0.05. In the Figures, three different levels of significance (p<0.05; p<0.01 and p<0.005) were considered.

## Results

### Axonal contact up-regulates RXR-γ in SCs

**Figure 1:**
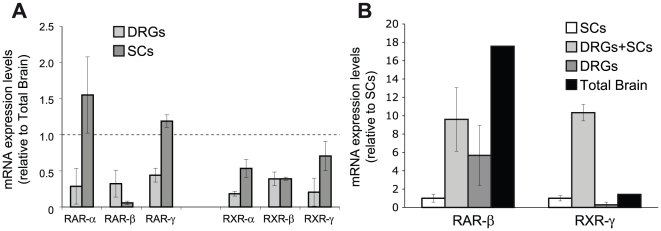
RXR-γ expression is increased in DRGN/SC cocultures. (**A**) The mRNA levels of all six RARs and RXRs were analyzed by Q-RT-PCR. The results are shown as the mean value ± SD relative to the levels found in whole adult rat brain (dashed line). (**B**) The mRNA levels of RAR-β and RXR-γ were analyzed by Q-RT-PCR in isolated SCs or myelination-competent DRGN/SC cocultures and compared to isolated DRG neurons and total adult rat brain. The results are shown as the mean value ± SEM relative to the levels found in isolated SCs.

We have recently shown that retinoic acid regulates myelin formation in the peripheral nervous system. In order to analyze which receptors were present in SCs, we performed real time RT-Q-PCR from primary Schwann cell cultures compared to primary DRG sensory neurons and total brain. In general, with the exception of RAR-β, all retinoid receptors, RARs and RXRs, were expressed by isolated Schwann cells at levels comparable or superior to DRG sensory neurons or total brain, which is known to express all retinoid receptors at relatively high levels (Fig. 1A).

Interestingly, not all retinoid receptors were homogenously maintained during the different stages of myelin formation or during sciatic nerve development [20]. This is especially notorious for RXR-γ, which was expressed at much higher levels in myelinating SC/DRGN cocultures than in isolated SCs or sensory neurons, in which it was present at very low levels (Fig. 1B). For comparison, RAR-β mRNA levels that were initially higher in DRGN than in SCs, remained relatively unchanged from DRGN cultures in myelination-competent SC/DRGN cocultures (Fig. 1B).

**Figure 2:**
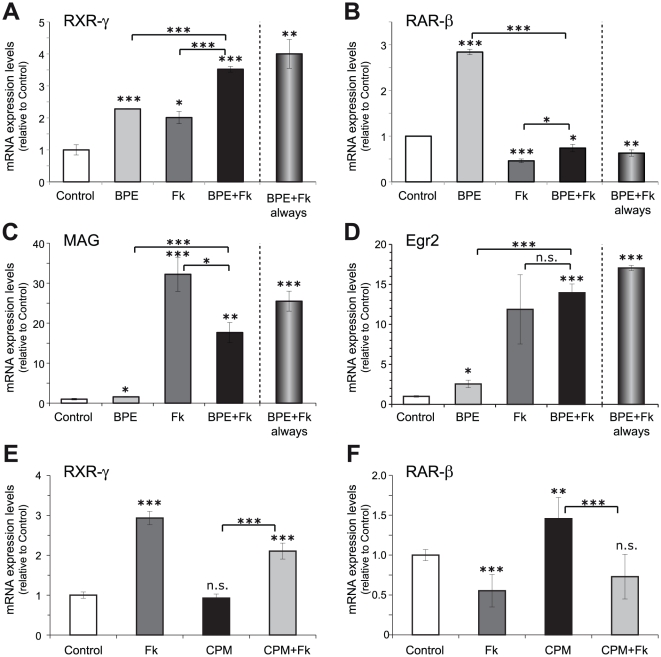
RXR-γ levels in SCs are induced by axonal contact mimickers. (**A**–**D**) Isolated SCs were adapted for 24 hours to the absence of the axonal contact mimickers BPE and forskolin and subsequently treated for 48 hours with 2 µM forskolin (Fk), 60 µg/ml BPE or the combination of both, and the levels of RXR-γ (**A**), RAR-β (**B**), MAG (**C**) and Egr2 (**D**) were analyzed by Q-RT-PCR. In all instances, the levels reached after the acute combined treatment were compared to SCs continuously grown in the presence of BPE and Fk (BPE+Fk always). (**E**–**F**) SCs were adapted for 24 hours to the absence the axonal contact mimickers and subsequently treated for 48 hours with 2 µM forskolin (Fk), 5 µM cyclopamine (CPM) or the combination of both, and the levels of RXR-γ (**E**) and RAR-β (**F**) were analyzed by Q-RT-PCR. All values are shown as the mean ± SD relative to their respective controls (cells grown in the presence of vehicle). Significance was assessed by unpaired Student's *t*-test. n.s.: non-significant; *: p<0.05; **: p<0.01; ***: p<0.005.

These results suggest that cell-to-cell contact upregulates RXR-γ expression, either in the SCs, in the DRGNs or in both cell types. As SCs express higher levels of retinoid receptors than DRGNs, and it is well known that neuronal contact strongly influence its physiology, we decided to analyze whether axonal contact could upregulate RXR-γ expression in SCs. DRGNs are pseudo-unipolar neurons; they present a bifurcated axon with central and peripheral branches and no dendrites. Axonal contact in SCs can be partially mimicked *in vitro* by the exposition to cAMP-elevating agents [31] such as the adenylate cyclase activator forskolin, especially in combination with the neuregulin glial growth factor (GGF). In order to ascertain whether, effectively, axonal contact is the responsible for the increased levels of RXR-γ mRNA observed in myelination-competent SC/DRGN cocultures, isolated SCs were treated with forskolin, both in the presence or absence of a GGF-enriched BPE. Treatment with either BPE or forskolin produced an increase in RXR-γ mRNA levels with a maximum effect obtained with the combination of both factors (Fig. 2A). On the other hand, BPE increased the low basal RAR-β mRNA levels, while forskolin produced a small but reproducible down-regulation that was even more apparent with the combination of both factors (Fig. 2B). As expected, myelin-associated glycoprotein (MAG) mRNA levels and Egr2, two markers of differentiated SCs, were highly up-regulated by forskolin, both in the presence or absence of BPE (Fig. 2C–D).

Although forskolin is used frequently to mimic axonal contact, it also influences Hedgehog (Hg) signaling. As several studies show cross-regulations during embryonic development between RA and Hg signaling pathways, we decided to analyze if forskolin treatment could be able to influence Hg signaling in SC cultures, which, in turn, might be responsible for changes in RAR-β and RXR-γ expression levels and not the mimicked axonal contact effect by forskolin. For this purpose, Hh signaling was blocked by treatment with the specific Hh receptor smoothened inhibitor cyclopamine, and the RAR-β and RXR-γ mRNA levels were determined. While treatment with forskolin alone produced an increase in RXR-γ and a reduction in RAR-β mRNA levels, cyclopamine treatment failed to mimic these effects (Fig. 2E–F). Cyclopamine did not affected RXR-γ mRNA levels whilst, contrary to the effect of forskolin, it did produce a small increase in RAR-β mRNA levels. In the presence of the smoothened inhibitor cyclopamine, forskolin was still able to increase RXR-γ mRNA levels and to decrease RAR-β mRNA levels. These results indicate that the Hh signaling is not involved in the effect of the axonal mimicker forskolin on SCs.

**Figure 3:**
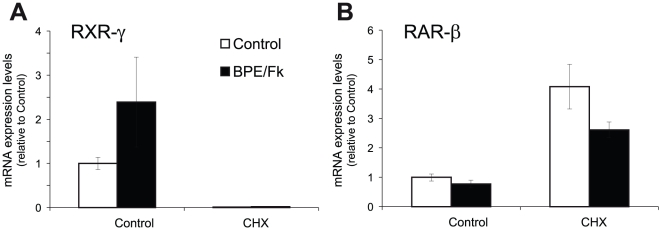
Protein synthesis inhibition abrogates RXR-γ, while increasing RAR-β mRNA steady state mRNA levels. SCs were treated with the axonal mimickers forskolin and BPE for 24 hours in the presence or absence of 10 µg/ml of the protein synthesis inhibitor cycloheximide (CHX), and RXR-γ (**A**) and RAR-β (**B**) mRNA levels were determined by Q-RT-PCR. All values are shown as the mean ± SEM relative to their respective controls in the absence of cycloheximide.

Next, we analyzed whether *de novo* protein synthesis was required for RXR-γ induction by axonal contact mimickers. Interestingly, the presence of the protein synthesis inhibitor ciycloheximide produced an almost complete suppression of RXR-γ steady state mRNA levels (Fig. 3A), which could not be reverted by the addition of BPE and forskolin. This result suggests that SCs express a labile inducer, whose continuous presence is required for maintenance of RXR-γ mRNA levels. On the other hand, RAR-β mRNA steady-state levels were increased in the absence of new protein synthesis, while the small reduction achieved by the combined treatment with BPE and forskolin was still visible although at a lesser extent (Fig. 3B).

All together, these results suggest that RXR-γ and RAR-β steady-state mRNA levels are maintained in normal conditions and regulated by axonal contact mimickers throughout different mechanisms.

### Retinoic acid differentially regulates RAR-β and RXR-γ mRNA levels in SCs

The fact that RA can modulate myelin formation in the PNS [20], together with the multiple descriptions of retinoids being able to regulate their own receptors levels in several cell types, led us to clarify whether the same could be true in myelinating Schwann cell/sensory neurons cocultures.

**Figure 4:**
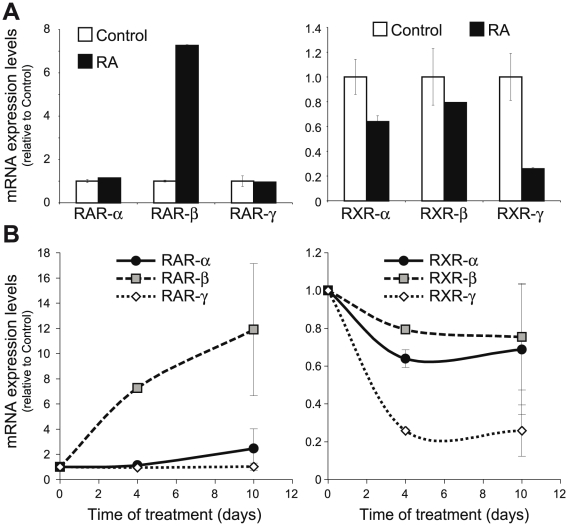
RA upregulates RAR-β and downregulates RXR-γ in myelinating cocultures. (**A**) DRGN/SC cocultures were allowed to myelinate for 7 days in the presence or absence of 1 µM RA, and the relative levels of RAR-α, -β and -γ, as well as RXR-α, -β and -γ were determined by Q-RT-PCR. All values are shown as the mean ± SD relative to their respective controls (sister cultures treated only with vehicle). (**B**) Myelinating cocultures were treated with 1 µM RA from the time of ascorbic acid induction and the levels of all six retinoid receptors were determined by Q-RTR-PCR at different times of treatment. All values are shown as the mean ± SD relative to their respective controls (myelinating cocultures treated only with vehicle for the same length of time).

Retinoic acid treatment produced a tremendous increase in RAR-β mRNA levels without affecting RAR-α or -γ mRNA levels in myelinating Schwann cell/sensory neurons cocultures (Fig. 4A). On the other hand, the retinoid produced a small but reproducible decrease in RXR-α and -β levels, at the same time that RXR-γ mRNA was highly down-regulated. This effect could already be seen after 4 days of treatment, a time point in which active myelin formation is taking place, as well as after 10 days of treatment in which myelin formation in control cocultures has already taken place almost completely (Fig. 4B).

**Figure 5:**
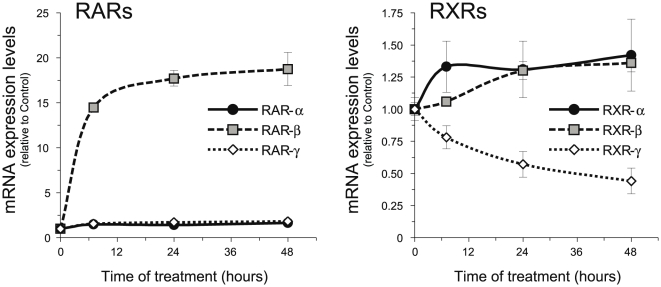
RA upregulates RAR-β mRNA and downregulates RXR-γ mRNA in isolated SCs. SCs were treated with 1 µM RA for the times indicated and the relative levels of all six retinoid receptors were determined by Q-RT-PCR. All values are shown as the mean ± SD relative to their respective controls.

Although retinoid receptors expression in SCs is generally higher that in isolated DRG neurons, the later also express them, so, at this moment, we could not know whether their regulation by RA in myelinating cocultures is mostly due to the SC component or if the neuronal levels are also changed. To ascertain if the variations seen in myelinating cocultures are due to a direct regulation of their mRNA levels in Schwann cells, we examined if RA could also regulate retinoid receptors in pure isolated primary Schwann cells cultures. As observed in myelinating cocultures, retinoic acid treatment increased RAR-β mRNA levels without affecting RAR-α and -γ levels (Fig. 5). Regarding RXRs, retinoic acid practically did not change RXR-α and -β mRNA levels, while RXR-γ was highly down-regulated, similarly to what occurred in myelinating cocultures.

**Figure 6:**
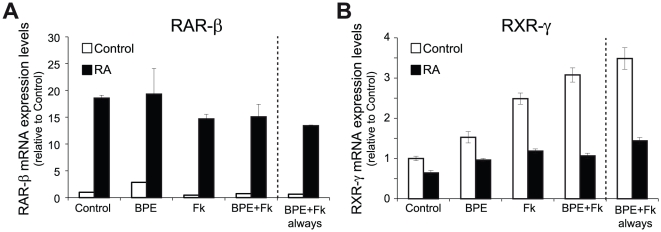
Influence of axonal contact mimickers on RXR-γ and RAR-β regulation by RA. RXR-γ downregulation is higher in the presence of axonal contact mimickers, while RAR-β upregulation was similar irrespective of the growth conditions. Isolated SCs were adapted for 24 hours to the absence of the axonal contact mimickers BPE and forskolin and subsequently treated for 48 hours with 1 µM RA in the presence or absence of 2 µM forskolin, 60 µg/ml BPE or the combination of both, and the mRNA levels of RAR-β (**A**) and RXR-γ (**B**) were analyzed by Q-RT-PCR. In all instances, the levels reached after the acute combined treatment were compared to SCs continuously grown in the presence of BPE and Fk (BPE+Fk always). All values are shown as the mean ± SEM relative to their respective controls.

As axonal contact was able to modulate retinoid receptors levels, and the above experiments depicted in Fig. 5 were performed with isolated SCs in the continuous presence of the axonal contact mimickers forskolin and BPE, we next asked whether RA-regulation of RAR-β and RXR-γ in SCs was dependent on the presence of these factors. As expected, RA produced a strong up-regulation of RAR-β mRNA levels irrespective of the presence of forskolin and/or BPE (Fig. 6A). Unexpectedly, RA downregulation of RXR-γ was at least partially dependent on the presence of the axonal contact mimickers. While RA was able to inhibit RXR-γ in the presence of the axonal mimickers BPE and forskolin, either alone or in combination, the retinoid was only able to slightly down-regulate the already diminished levels found in the absence of axonal mimickers (Fig. 6B). Analyzing the results from a different perspective, in the presence of RA, neither BPE nor forskolin, either alone or in combination, were able to upregulate RXR-γ mRNA levels.

### RAR-β and RXR-γ are regulated by a RAR-dependent mechanism

**Figure 7:**
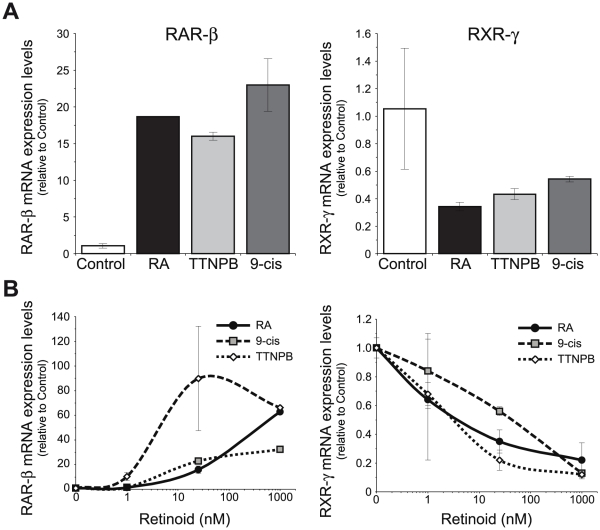
RAR-β and RXR-γ regulation is mediated by RAR. (**A**) Myelinating cocultures were treated for 7 days with 1 µM of the natural retinoids *all-trans*-retinoic acid (RA) and *9-cis*-retinoic acid (*9-cis*), or the synthetic compound TTNPB, and the relative mRNA levels of RAR-β and RXR-γ were determined by RT-Q-PCR. (**B**) Isolated SCs were treated for 48 hours with different doses of *all-trans*-retinoic acid (RA), TTNPB or *9-cis*-retinoic acid (*9-cis*), and the relative levels of RAR-β and RXR-γ mRNAs were determined by RT-Q-PCR. All values are shown as the mean ± SD relative to their respective controls.

As RA actions can be mediated by both RAR and RXR, and both groups of receptors have been shown to be active during myelin formation in Schwann cells [20], we asked which receptor(s) mediate RAR-β and RXR-γ regulation. Myelinating cocultures were treated for seven days with the natural retinoid *all-trans*-retinoic acid, which binds to RAR as well as to RXR although with a lower affinity, as well as with *9-cis*-retinoic acid that binds with similar affinity to RAR and RXR, or with the pure RAR synthetic agonist TTNPB that only activates RAR. All three retinoids were equally able to induce RAR-β and down-regulate RXR-γ mRNA levels to a similar extent, which indicates that binding to RAR is sufficient to mediate the effect of retinoic acid (Fig. 7A). All three retinoids were also able to regulate to a similar extent both genes in purified primary Schwann cell cultures (Fig. 7B), although TTNPB was more potent, specially regarding RAR-β up-regulation, indicative of its higher affinity for RAR binding compared to the natural retinoids [32]. At the low concentration of 1 nM, TTNPB already produced a statistically significant down-regulation of RXR-γ and upregulation of RAR-β, while *9-cis*-RA or *all-trans*-RA only produced a small regulation of both receptors that was not statistically significant. At the more elevated but still physiologically relevant dose of 25 nM, all three retinoids were able to significantly regulate RAR-β and RXR-γ gene expression.

### RAR-β and RXR-γ are regulated at the transcriptional level

Next, we sought to find whether the regulation of RAR-β and RXR-γ steady-state mRNA levels by RA is due to a transcriptional mechanism or if, alternatively, other mechanisms such as changes in mRNA stability were involved. Hetereogeneous nuclear RNAs or hnRNAs are the precursors of the mature mRNAs still present on the nucleus of the cell. They present very short half-lives, being processed as they are exported to the cytoplasm. Measuring their levels constitutes a good estimation of the transcription rate for a given gene [33]. In order to ascertain if the transcription rates for RAR-β and RXR-γ were regulated by retinoic acid, myelinating cocultures were treated with the hormone for 1, 5 or 14 days and the amount of newly synthesized mRNAs was determined by measuring the hnRNA levels.

**Figure 8:**
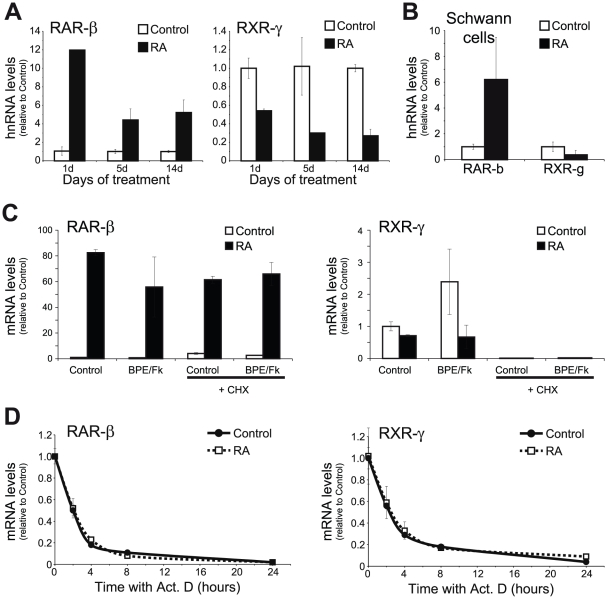
Transcriptional regulation of RAR-β and RXR-γ by RA. (**A**) The levels of newly synthesized hnRNA for RAR-β and RXR-γ were determined by RT-Q-PCR in myelinating cocultures treated with 1 µM RA for the indicated times. All values are shown as the mean ± SD relative to their respective controls. (**B**) Newly synthesized RAR-β and RXR-γ hnRNA was determined in isolated SCs treated or not with 1 µM RA for 48 hours. All values are shown as the mean ± SD relative to their respective controls. (**C**) SCs were treated with 1 µM RA in combination or not with the axonal mimickers forskolin and BPE for 24 hours in the presence or absence of 10 µg/ml of the protein synthesis inhibitor cycloheximide (CHX), and RAR-β and RXR-γ mRNA levels were determined by Q-RT-PCR. All values are shown as the mean ± SEM relative to their respective controls in the absence of cycloheximide and retinoic acid. (**D**) Isolated SCs were pretreated or not with 1 µM RA for 24 hours and the stability of RAR-β and RXR-γ mRNA was determined by incubating the cells with 10 µg/ml actinomycin D for varying times in the continuous presence or not of the retinoid. All values are shown as the mean ± SD relative to their respective time zero. RA-treated cells presented RAR-β mRNA levels 59 times higher than control cells, while RXR-γ levels were only 40% of those from control cultures.

Retinoic acid produced a strong increase in RAR-β hnRNA levels already at day 1 of treatment, an increment that was maintained, although at a lower level, for the two-weeks time point analyzed (Fig. 8A). Conversely, RXR-γ hnRNA levels were already reduced after 1-day treatment with the retinoid, reduction that was maintained and even exacerbated during the whole time period analyzed. Similarly, in isolated SCs, treatment with the hormone for 48 h produced an increase in RAR-β hnRNA, whilst down-regulating RXR-γ hnRNA levels (Fig. 8B).

As retinoids can regulate gene expression both directly, through binding of their receptors to the promoters of their regulated genes, as well as indirectly, by regulating other transcription factors which, in turn, are the final mediators of the retinoid actions, we decided to analyze if RA regulation was dependent on protein synthesis. While RA was equally able to increase RAR-β mRNA in SCs both in the presence or absence of the protein synthesis inhibitor ciycloheximide, the already quite low levels of RXR-γ found in the presence of the inhibitor were not further down-regulated by RA (Fig. 8C), indicating that the later required newly synthesized proteins to be effective.

Although these results indicate that both genes are regulated by a transcriptional mechanism, they do not rule out the possible existence of an additional layer of regulation at the post-transcriptional level. To discern this, primary Schwann cells cultures were pre-treated with retinoic acid for 24 hours, followed by the addition of the RNA polymerase inhibitor actynomicin D for different times, and the remaining mRNA levels of RAR-β and RXR-γ were analyzed by real time RT-Q-PCR. Although mRNA levels were quite different in the presence or absence of retinoic acid (59-fold higher for RAR-β and 2.5-fold lower for RXR-γ), their half-lives were identical in both circumstances (Fig. 8D), indicating that RAR-β and RXR-γ are regulated by retinoic acid exclusively at the transcriptional level.

### RXR-γ protein levels are regulated by retinoids

**Figure 9:**
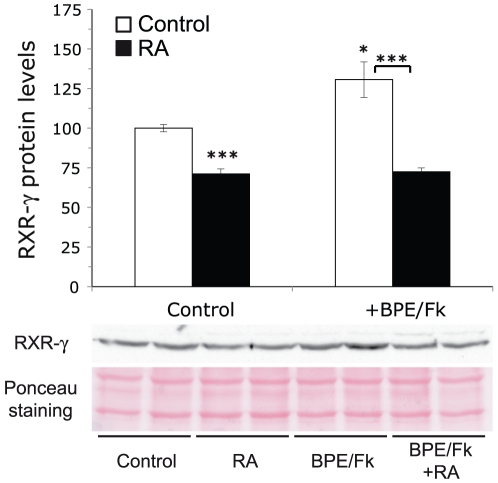
Retinoic acid and axonal contact mimickers regulate RXR-γ protein levels. Isolated SCs were adapted for 24 hours to the absence of the axonal contact mimickers BPE and forskolin and subsequently treated for 48 hours with 1 µM RA in the presence or absence of 2 µM forskolin and 60 µg/ml BPE, and the protein levels of RXR-γ were analyzed by western blot. The upper panel depicts the quantitation of the blots. The data are presented as the mean value ± SEM referred as the percentage over the controls. Significance was assessed by unpaired Student's *t*-test. *: p<0.05; ***: p<0.005.

Finally, we sought to analyze if the changes in RXR-γ steady state mRNAs levels by RA and axonal contact translate into increased receptor abundance at the protein level. Similarly to what occurs at the mRNA level, RA treatment of isolated SCs produced a small decrease in RXR-γ protein levels in the absence of axonal contact mimickers. The combined treatment with BPE and forskolin produced an increase in RXR-γ protein levels, which was prevented by treatment with the retinoid (Fig. 9).

## Discussion

Previous work from our lab and others has shown that retinoids play an important role during Schwann cell development and function. The results shown in this work demonstrate that retinoid receptors are indeed present in Schwann cells and that their expression levels are tightly regulated by retinoic acid itself as well as by agents that mimic axonal contact.

It has been described that retinoic acid signaling components are present in the peripheral nervous system and that after sciatic nerve injury there is an upregulation in retinoic acid signaling as measured by a RARE reporter in a transgenic reporter mouse [10]. This increased RA-signaling is accompanied by a transient upregulation of RAR-α, -β and -γ as well as RXR-α, but not of RXR-β and -γ, in cells morphologically resembling Schwann cells, although the presence of other cell types (i.e., macrophages) that also express high retinoid receptors levels, makes this deduction less conclusive. Similarly, retinoids play an important role during normal Schwann cell development and function, as regulators of myelin formation throughout two distinct mechanisms, one RAR-dependent that involves MAG down-regulation, and another one mainly RXR-dependent involving Egr2 upregulation [20]. This phenomenon is accompanied by the transcriptional regulation of the final effectors, the retinoid receptors. Especially interesting is RXR-γ, which is highly upregulated in myelination-capable sensory neuron/Schwann cell cocultures during the phase of Schwann cell proliferation and migration. Our results suggest that this increase most probably is dependent on axonal contact and occurs in the glial component of the coculture, because can be equally observed in isolated SCs in the absence of sensory neurons by the combined treatment with forskolin and BPE. Forskolin is an activator of adenyl cyclase that is a frequent component in Schwann cell cultures as cAMP is an important secondary messenger for their growth and maturation that mimics several of the effects induced by axonal contact [31]. A G-protein coupled receptor Gpr126 has been recently identified to regulate an *in vivo* surge of cAMP in Schwann cells, which allows for Oct 6 activation at the promyelinating stage [34]. On the other hand, BPE, also routinely used in Schwann cell cultures, is a supplement rich in several growth factors such as neuregulins, bFGF and PDGF that are important for promoting the survival of Schwann cell precursors [35]. Together with the elevation of cAMP levels mimicking axonal contact, these growth factors strongly influence SC physiology and have a synergistic effect on Schwann cell proliferation [36]. Several studies show cross-regulations during embryonic development between the RA and Hedgehog signalling pathway [37] while Desert Hedgehog is also known to be secreted by SCs and their precursors regulating the formation of the perineurium and the maintenance of the nerve, including myelinated fibers [38], [39]. Forskolin increases cellular cAMP levels by direct stimulation of adenylyl cyclase which in turn results in the activation of protein kinase A (PKA) [40], [41]. On the other hand, activated PKA inhibits Hh signaling [42], [43], which opens the possibility that the observed RAR-β and RXR-γ regulation by forskolin could be dependent on Hh signaling. The fact that blocking the Hh receptor smoothened by the specific inhibitor cyclopamine was not able to mimic the effect of forskolin, and, in the case of RAR-β, even modulate its expression in the opposite direction, indicates that Hh signaling is not participating on the regulation of RAR-β and RXR-γ expression by cAMP.

Schwann cell behavior depends on the combination of extrinsic growth factors and hormones along with juxtacrine signaling coming from the neurons. A clear example is the induction of Egr2, the major myelinogenic transcription factor, whose expression depends mainly on axonal contact [44], [45] but can also be regulated by extrinsic factors like retinoic acid [20]. Interestingly, Egr2 upregulation by RA is mostly dependent on RXR activation, whose levels are also upregulated in Schwann cells by axonal contact. It is tempting to speculate that the same or similar regulatory mechanisms that control Egr2 expression in Schwann cells could also be regulating RXR-γ expression. Another intriguing possibility is that Egr2 upregulation by axonal contact could be partially dependent on RXR-γ activation, either by retinoids themselves or by other agents capable of activating this receptor, like docosahexaenoic acid, a long-chain polyunsaturated fatty acid that is highly enriched in the adult mammalian brain [46] as well as in sciatic nerve during myelin formation [47] and has been shown to enhance neurite formation in DRG sensory neurons [48].

On the other hand, retinoids themselves are also potent regulators of their own receptors in Schwann cells. RAR-β is highly upregulated by RA in a similar fashion to what has been described in other cell types, including PC12 cells [6], neuroblastoma cells [24], human B- and T-cells [23] or *in vivo* in neural crest-derived cells of chick embryos [25]. Presumably, this is a direct regulation mediated by the RAREs present in the minimal promoter of this gene [49]. The upregulation of RAR-β by RA is one of the transcriptional regulations more conserved throughout evolution and, in fact, even in the amphioxus, the closest living invertebrate relative of the vertebrates, in which a single RAR gene (*AmphiRAR*) exists, retinoic acid is able to induce its expression in the nerve cord during development [50].

Interestingly, although cAMP has been described to be able to synergize with the induction of RAR-β by RA in embrionary carcinoma cells [51], we cannot observe this synergism in Schwann cells, in which maximum activation by the retinoid is already achieved in the absence of cAMP activators which, if any, have a negative impact on RAR-β expression levels, in a similar manner to what has been described in neuroblastoma cells [24].

Much less is known about the control of RXR expression levels by retinoids. The down-regulation of some RXR-γ transcripts by retinoic acid during differentiation of F9 mouse teratocarcinoma cells has already been described [52], although the mechanisms involved are quite different from the effect of RA on Schwann cells. In F9 cells, the effects of the retinoid are not dependent on *de novo* protein synthesis, while in Schwann cells, the presence of the protein synthesis inhibitor cycloheximide not only abolishes RXR-γ regulation but, in fact, it produces a strong decrease in mRNA levels, uncovering the existence of a labile activator of RXR-γ expression in Schwann cells. Additionally, this *de novo* protein synthesis dependence indicates that the effect of the retinoid is not due to the direct binding of RAR on RXR-γ promoter region. In line with this, no putative RARE can be found in the promoter sequences upstream of RXR-γ rat gene. Unfortunately, neither the upregulation by axonal contact mimickers nor the downregulation by RA can be found in promoter assays with constructs that compass up to 3 Kb upstream of the transcription starting site of rat RXR-γ gene (data not shown).

RXR-γ expression is developmentally regulated during peripheral nervous system development in rodents [53]. While in chicken embryos RXR-γ is expressed from the onset of neural crest migration [54], rat RXR-γ expression is not detectable in dorsal root and trigeminal ganglia until 14.5 days post coitum, suggesting a role for this nuclear receptor in DRGs of late embryos. The *in situ* hybridization technique does not allow discrimination on the nature of the cell types that express RXR-γ in the ganglia, although the same authors already reported a comparatively very high RXR-γ expression level in 7-day-old rats. The detection onset of RXR-γ in these ganglia coincides with the period during which they begin to innervate their peripheral and central targets. Interestingly, until 10.5 dpc, Egr2 expression in peripheral nerves is restricted to the proximities of the ganglia, but at 15.5 there is a dramatic activation of Egr2 expression being reported along the whole sciatic nerve [55]. This stage corresponds precisely to the period of the transition from precursors to embryonic Schwann cells [56], a transition that involves the activation of *Egr2* [44]. Interestingly, RXR-γ signaling has just been shown to be involved in oligodendrocyte differentiation and to accelerate CNS remyelination [57].

RXR belongs to the Class II subfamily of nuclear receptors and, besides being able to act by itself as a ligand-dependent transcription factor, its major role is as the heterodimerizing partner of the Class I subfamily of nuclear receptors, which is formed by RAR, as well as the vitamin D3 (VDR), the thyroid hormone (TR) or the peroxisome proliferator activated receptors (PPAR) [21]. This central role as a required partner in nuclear receptor signaling implies that the regulation of RXR levels will be relevant not only for its own signaling, but also for regulating the ability of SCs to respond to a vast array of hormones, some of which are likely to play an important role in SC physiology. Further studies need to be conducted in order to determine whether RXR-dependent regulation of myelination is mediated by homodimers or if it is acting just as a heterodimerizing partner for other nuclear receptors. Regarding this, only RXR-α null-mice are not viable and show a clear phenotype. In fact, even the triple compound RXR-α^+/−^/RXR-β^−/−^/RXR-γ^−/−^ mutant mice are viable and almost indistinguishable from RXR-α^+/−^ mice [58], suggesting that all three isoforms are basically interchangeable, making more difficult to study the involvement of RXR on myelin formation *in vivo*. Animal models of forced overexpression after birth, as well as conditional knock-outs of the different RXR isoforms on Schwann cells should provide further mechanistic insights into the role of RXRs on Schwann cell differentiation and subsequent peripheral myelin formation.

## Supporting Information

Table S1 Q-RT-PCR primer list.
(DOC)
