# Supplementary material for: Regulation of Retinoid Receptors by Retinoic Acid and Axonal Contact in Schwann Cells
Source: PLoS One. 2011 Feb 28;6(2):e17023. doi: 10.1371/journal.pone.0017023 (PMC3046125; doi:10.1371/journal.pone.0017023)
Supplement: Table S1 — Q-RT-PCR primer list. (DOC) [file pone.0017023.s001.doc]

| **Gene** | **Orientation** | **Sequence (5’🡪 3’)** |
| --- | --- | --- |
| RAR-α | Forward | AGA TGG ACT GAC CCT GAA CCG |
|  | Reverse | CCA CCT TGT CTG GCT GCT CC |
| RAR-β | Forward | CAA TGC TGG CTT CGG TCC TC |
|  | Reverse | CTC AAG GTC CTG GCG GTC TC |
| RAR-γ | Forward | ACC CCC ACC ACC TCC TCG |
|  | Reverse | GCG TCT GAA GAA GCC CTT GC |
| RXR-α | Forward | TCA ATG GCG TCC TCA AGG TTC |
|  | Reverse | TGT CAC GGC AGG TGT AGG TCA G |
| RXR-β | Forward | GGA TTC CCG AAG CCC AGA C |
|  | Reverse | GCA TTG GAG GTG GAG GTG C |
| RXR-γ | Forward | GGG AGC GAG CAG AGA GTG AGG |
|  | Reverse | GTG GGG GAT GCG TTT GGC |
| MAG | Forward | TTC CAG GGA GCA CAC CGA C |
|  | Reverse | TGT AGC AGA CAA TGG CAA TCA GG |
| Krox20 | Forward | TGC CTG ACA GCC TCT ACC CG |
|  | Reverse | TCT CCA GCC ACT CCG TTC ATC |
| CypA | Forward | TGG CAA ATG CTG GAC CAA AC |
|  | Reverse | TTC CTG GAC CCA AAA CGC TC |
| 18S rRNA | Forward | GTA ACC CGT TGA ACC CCA TT |
|  | Reverse | CCA TCC AAT CGG TAG TAG CG |
| RAR-β hnRNA | Forward | TGG GTA AAT ACA CCA CGG TAA GAG C |
|  | Reverse | AAA TCA CCA AGA GGA GAC ACA AGC |
| RXR-γ hnRNA | Forward | TAC ACA GAT ACC CCA GTG AGT GCC |
|  | Reverse | GCC TCG GAG AAA CAT AAC CTA CC |
